# Supplementary material for: A deep learning AI model for determining the relationship between X-Ray detectors and patient positioning in chest radiography
Source: PLoS One. 2025 Oct 13;20(10):e0333209. doi: 10.1371/journal.pone.0333209 (PMC12517491; doi:10.1371/journal.pone.0333209)
Supplement: Attachment — Submitted filename: Response to Reviewers.docx [file pone.0333209.s002.docx]

**Dear Editor and Reviewers of the esteemed journal,**

**Greetings!**

**We appreciate the acknowledgment of our work by the editorial team and reviewers, and we have found your comments highly beneficial. We sincerely believe that these suggestions will help us present our research results and academic viewpoints more effectively. Below are our detailed responses to the revisions made to the manuscript. Please allow us to highlight our responses in distinct colors for quick reference.**

**Journal Requirements:**

**1. When submitting your revision, we need you to address these additional requirements.**

**Please ensure that your manuscript meets PLOS ONE's style requirements, including those for file naming. The PLOS ONE style templates can be found at**

**https://journals.plos.org/plosone/s/file?id=wjVg/PLOSOne_formatting_sample_main_body.pdf and https://journals.plos.org/plosone/s/file?id=ba62/PLOSOne_formatting_sample_title_authors_affiliations.pdf**

**Response: Revisions have been made according to the official templates provided, although minor discrepancies may exist due to the PDF format. We are happy to revise further if needed.**

**2. Please note that PLOS ONE has specific guidelines on code sharing for submissions in which author-generated code underpins the findings in the manuscript. In these cases, we expect all author-generated code to be made available without restrictions upon publication of the work. Please review our guidelines at https://journals.plos.org/plosone/s/materials-and-software-sharing#loc-sharing-code and ensure that your code is shared in a way that follows best practice and facilitates reproducibility and reuse.**

**Response: Author information has been revised according to the format.**

**3. We note that the grant information you provided in the ‘Funding Information’ and ‘Financial Disclosure’ sections do not match.**

**When you resubmit, please ensure that you provide the correct grant numbers for the awards you received for your study in the ‘Funding Information’ section.**

**Response: We confirm that the funding grant numbers are accurate, and intergovernmental certification has been provided.**

**4. We note that your Data Availability Statement is currently as follows: [All relevant data are within the manuscript and its Supporting Information files.]**

**Please confirm at this time whether or not your submission contains all raw data required to replicate the results of your study. Authors must share the “minimal data set” for their submission. PLOS defines the minimal data set to consist of the data required to replicate all study findings reported in the article, as well as related metadata and methods (https://journals.plos.org/plosone/s/data-availability#loc-minimal-data-set-definition).**

**Response: We confirm that the results related to the content of the article are included in the manuscript. As for the specific dataset used to train the model, it is not a public dataset and is too large to provide at present. Interested readers may contact jiangbo@csu.edu.cn for inquiries.**

**5. PLOS requires an ORCID iD for the corresponding author in Editorial Manager on papers submitted after December 6th, 2016. Please ensure that you have an ORCID iD and that it is validated in Editorial Manager. To do this, go to ‘Update my Information’ (in the upper left-hand corner of the main menu), and click on the Fetch/Validate link next to the ORCID field. This will take you to the ORCID site and allow you to create a new iD or authenticate a pre-existing iD in Editorial Manager.**

**Response: We have completed the association of the ORCID iD as required.**

**6. Please ensure that you include a title page within your main document. You should list all authors and all affiliations as per our author instructions and clearly indicate the corresponding author.**

**Response: A new title page has been uploaded for your review.**

**We thank the reviewers for their thorough suggestions, which prompted us to reflect deeply on our manuscript. Initially, we did not elaborate on why we established such a model; in fact, our research aims to develop a fully automatic chest X-ray machine that can significantly reduce the workload of radiological technicians in populous countries. Especially in medical examination centers where many examinees require long waiting times, and in infectious disease zones or warfronts, automated examinations can help avoid various issues. The five-category classification of the model is designed as a critical decision node for this automation. We have continuously optimized this aspect of our work, and based on the reviewers' comments, we have incorporated recent progress into the manuscript to better conform to academic paper standards and form robust comparisons. Accordingly, we have extensively revised and optimized the dataset, experimental design, and statistical analysis. The following are detailed responses to the individual issues raised.**

**Reviewer #1: General:**

**The introduction needs to be reworked. There are some irrelevant details (see comments) and some missing information. For instance, why no background on CNN or more detailed discussion of prior image classification work? Also, the purpose/approach of the study should be clarified.**

**Response: The introduction has been rewritten, detailing why the model was established, the rationale behind the chosen classifications, and how our work builds upon previous studies while explaining the reasons for adopting this method.**

**Was there any attempt to measure distance to participants or otherwise quantify their position in space? This seems important and is not addressed.**

**Response: Apologies for not detailing this in the initial submission. Our spatial distances strictly adhere to the imaging methods described in literature, both in terms of shooting distance and positioning techniques, which are standard practices in China for chest X-rays.**

**What are other alternatives to this approach? I think this paper is missing any comparison to current work. Pose estimation is quite good these days and there are likely off-the-shelf products that could aid in real-time positioning of a patient. One could even imagine a simple edge detection algorithm could be used here. Your approach is reasonable in a vacuum, but the paper is missing justification and context, especially considering that the only measure used is whether or not the patient is "too high" or "too low".**

**Response: Following your suggestion, we have added a detailed discussion in the first paragraph of the Discussion section regarding alternative approaches, why AI was chosen over other positioning methods, and the criteria for measurement.**

**The results section is limited. It seems to be missing a reference to the Grad-CAM results. There is nothing on the training or validation of the model. I would be interested in seeing some of the testing that went into selecting your hyperparameters too.**

**Response: As suggested, descriptions and discussions of Grad-CAM have been added.**

**I would include baseline example images of each category. Figure one has images that are unlabeled, and it is not clear what is what.**

**The figures in general seem a bit blurry and do not have good/descriptive legends.**

**I have somewhat of a problem with the simple categories. They are very limited. What is too high? What is too low? How is that actually determined (outside of expert opinion)? What about distance from patient? Posture? Etc. Is that the only issue that is present? What if someone is just a little too high or a little too low? These categories are presented without good context. I also wonder if your categories correlate at all with end image quality (the actual x-rays). This is very important I think for practical application and it is not addressed here.**

**Response: Your points are well taken. We have strict reference standards that Chinese radiological technicians follow. "Too high" and "too low" refer to positions relative to the correct method, which stipulates that the detector's upper edge must be 3cm away from the examinee's acromion on both sides. This ensures batch processing and standardization of chest X-rays.**

**Specific:**

**Lines 36 – 40: Why do you mention fluoroscopy here? I don’t see how this section fits into the introduction. Fluoroscopy is a separate technique that would not necessarily be used in place of chest x-ray (or vice versa). I don’t see the reason for the comparison. This section does not add anything of value. I would suggest removing it or rewriting it to better integrate with the paper. I would rather see background about where and how x-ray might be used.**

**Response: Following your suggestion, we have removed the content related to fluoroscopy. Initially, we compared chest examination methods before the advent of DR as a common practice in large populations. However, with rapid technological development, comparing fluoroscopy no longer holds much relevance.**

**Lines 41-48: This feels like a better start to the introduction and is more relevant to the research question. I would consider integrating this paragraph and the first to improve flow.**

**Response: We have thoroughly integrated the introduction and discussion sections to improve the flow.**

**Lines 45-48: I would like to see some citations here. Has there been prior work done? Why is this an issue? Please expand more here.**

**Response: We have added references to similar studies as per your suggestion. Our CNN architecture was designed based on one of the highly feasible networks from previous studies, although papers exactly matching our model's application are scarce.**

**Lines 55-59: I think that you need to clarify that this modeling is attempting to use external camera data to aid in positioning. It is not obvious.**

**Response: We have specifically described in the Methods section that the data originates from scene videos captured by a camera fixed above the X-ray tube.**

**Lines 83 – 84: Was the classification solely based on visual distance/positioning? I would think that the resulting x-ray should be the standard here i.e., that the images are graded based on the clarity/positioning/quality of the end result image. Why was this not done?**

**Response: Your point is well taken. We adhere to strict reference standards followed by Chinese radiological technicians. "Too high" and "too low" refer to positions relative to the correct method, which stipulates that the detector's upper edge must be 3cm away from the examinee's acromion on both sides, aiding in batch processing and standardization of chest X-rays.**

**Line 87: Please specify what the “No-one” and “Others” classifications mean.**

**Response: In the introduction, we have added descriptions. The model provides visual recognition for an automated shooting machine under development. If the examinee has not reached the detector area, video and voice prompts guide them. Therefore, detecting the absence of a person ("No-one") and identifying incorrect postures ("Others") are crucial for guiding proper positioning.**

**Lines 94-95: Why such a comparatively small test set?**

**Response: Taking your advice, we have reallocated the dataset according to similar studies, ensuring the test set comprises 1/5 of the total data.**

**Line 99: How is this different from conventional models? Are you referring to a particular architecture? A CNN with 2 convolutional layers and 3 fully connected layers does not strike me as unusual.**

**Response: Our customized CNN architecture is modeled after previous studies because this configuration has achieved high accuracy in judging standing postures.**

**Line 115: I am surprised by this low batch size producing the best results. It isn’t unreasonable, it is just lower than usual (for a “low” batch size).**

**Response: Using the smallest batch size aimed to maximize the effectiveness of the limited training set. We verified that batch sizes between 1 and 5 showed no significant changes in accuracy, so using a batch size of 5 can expedite training and hyperparameter tuning.**

**Lines 132-134: What was the result here? Did you make any changes to the model? The referenced figure is interesting, but it isn’t obvious to me what you did with that info.**

**Response: We have detailed statistical processing and included K-fold cross-validation, McNemar’s test for statistical differences, confusion matrices, calibration curves, and Brier scores.**

**Lines 154-156: It seems that this is repeated from table 1. I do not think the information needs to be in both places. The table doesn’t really add much. Additionally, it is confusing to read, at a minimum I would add acronyms to the legend.**

**Response: We have used K-fold validation to manage data and clarified three tables to present the data succinctly.**

**Line 158: Why is this section not in the intro? I don’t think it belongs in the discussion.**

**Response: We have comprehensively integrated the introduction and discussion sections.**

**Line 169: This entire subsection is a mishmash of information that belongs elsewhere. The Grad-CAM results should be in the results section. The methods should mention that you tested multiple configurations of convolutional layers. It is not clear what is considered a well-positioned x-ray.**

**Response: We have added discussions on Grad-CAM and introduced an experimental group with residual networks, finding that our customized CNN offers better interpretability.**

**Reviewer #2:**

**Your manuscript is technically strong and provides valuable data to support its conclusions. However, there are a few areas where refinements could further enhance the clarity, rigor, and practical relevance of the work.**

**Response: Thank you for acknowledging our work. We have added new experimental groups, restructured the dataset, and employed the recommended statistical methods to evaluate the model's performance.**

***Model validation**

**The reported accuracy metrics are impressive, but the validation and test sets are relatively small compared to the training data. It would be helpful to discuss whether the results are generalizable to broader populations or different imaging setups. Additionally, consider addressing how the model might perform in varied real-world scenarios, such as with different equipment or patient positions, to better illustrate its robustness.**

**Response: Following your advice, we have reallocated the test set, ensuring 1/5 of the data is used for testing. We also increased testing across various categories and discussed the model's generalizability.**

***Statistical analysis**

**Your study employs solid and appropriate statistical methods, but there are a few areas where additional details could enhance the clarity and reliability of the results.**

**Confidence Intervals (CIs): Including confidence intervals for key performance metrics like accuracy, sensitivity, specificity, and precision would provide a clearer picture of their consistency and reliability. Confidence intervals offer insight into potential variability and allow readers to assess the robustness of findings in different scenarios.**

**Class imbalance: The imbalance in image categories, such as "Too Low," could potentially affect the model’s performance metrics. To address this, consider reporting class-specific metrics, such as precision, recall, and F1-scores for each category. This will highlight any discrepancies and ensure transparency regarding underrepresented categories.**

**Statistical comparisons: If comparing the model's performance to other baseline methods or alternative approaches, including statistical significance testing would be beneficial. For example, using McNemar’s test for paired categorical data can demonstrate meaningful performance differences.**

**Model calibration: Providing an evaluation of model calibration, such as a calibration curve or Brier score, would show how well predicted probabilities align with actual outcomes, critical in clinical applications.**

**Variability assessment: Techniques like bootstrapping or cross-validation can assess metric variability across different data subsets, offering a comprehensive view of generalizability and reliability.**

**Response: We have added experimental groups and recalibrated all models. Statistical analyses now include McNemar’s tests, confusion matrices, calibration curves, and Brier scores. We have also clarified the criteria for "Too High" or "Too Low" and addressed the imbalance in "Too Low" data, reflecting the original scenario's requirements.**

***Presentation and Writing**

**Overall, the manuscript is well-written, but some sections, particularly the methodology, feel overly dense. Simplifying the language and structure would make the content more accessible. Captions for Figure 3 (training process) and Figure 4 (Grad-CAM) could be expanded for better explanation.**

**Response: We have simplified the presentation of experimental results and statistical data, using three tables to clearly present complex outcomes. We also added comparisons of Grad-CAM between experimental and control groups and included interpretability discussions in the Discussion section.**

***Discussion of limitations**

**The discussion does not fully explore some limitations, such as the imbalance in image categories, which may affect model performance. Addressing this and proposing strategies for handling imbalances in future research would be beneficial.**

**Response: We have explained the "Too Low" data issue and moderately increased training samples. More limitations are discussed, noting that current improvements are insufficient for complete automation without human intervention.**

***Future directions and practical implications**

**Expanding on practical implications, such as reducing the workload of radiologic technologists, by discussing challenges like ethical considerations and user training, would add depth to the discussion. This would help readers understand real-world clinical implementation.**

**Response: We have elaborated on why we established this model and the changes it aims to bring. This visual recognition model supports a fully automated chest X-ray machine, aiming to significantly reduce the workload in heavily populated countries.**

**Thank you for your patience and thorough review. Any further questions can be directed to jiangbo@csu.edu.cn.**

**Best regards,**

**Your closest Situ**

**January 18, 2025**
