# Supplementary material for: A deep learning AI model for determining the relationship between X-Ray detectors and patient positioning in chest radiography
Source: PLoS One. 2025 Oct 13;20(10):e0333209. doi: 10.1371/journal.pone.0333209 (PMC12517491; doi:10.1371/journal.pone.0333209)
Supplement: Attachment — Submitted filename: Response to Reviewers2.docx [file pone.0333209.s003.docx]

**Dear Editor and Reviewers of the esteemed journal,**

**Greetings!**

**We appreciate the acknowledgment of our work by the editorial team and reviewers, and we have found your comments highly beneficial. We sincerely believe that these suggestions will help us present our research results and academic viewpoints more effectively. Below are our detailed responses to the revisions made to the manuscript. Please allow us to highlight our responses in distinct colors for quick reference.**

**Reviewer #3: Thank you for the opportunity to review your manuscript titled "A Deep Learning AI Model for Determining the Relationship Between X-Ray Detectors and Patient Positioning in Chest Radiography." Your work addresses a highly relevant and timely topic, especially as AI continues to influence the optimisation of imaging protocols and the improvement of diagnostic quality in medical imaging.**

**The manuscript presents a promising application of deep learning to assess patient positioning in relation to X-ray detector alignment a factor critical for image quality and diagnostic accuracy. The use of AI to provide potential decision support or automated feedback to radiographers has clear clinical implications, particularly in standardising chest radiography practices and minimising repeat exposures.**

**Strengths of the manuscript include:**

**-A well-motivated research problem with clear clinical relevance.**

**-Appropriate use of deep learning methodology.**

**-Potential for integration into clinical workflows for quality assurance or education.**

**However, there are a few areas that could benefit from further clarification or development:**

**1.Model Explainability: Consider expanding on how the model's outputs (e.g., heatmaps or classification scores) are interpreted in the context of positioning accuracy.**

**Response: We thank the reviewer for highlighting model interpretability. We have expanded the explanation of Grad-CAM outputs in the Results section (Fig 4) and added a dedicated analysis in the Discussion (Lines 305-318). The heatmaps demonstrate that Model A’s attention aligns with clinical positioning criteria, while Model B’s residual structure prioritizes performance over visual interpretability, both offering complementary advantages for clinical deployment.**

**2.Data Description: Please provide more detail regarding the dataset used, such as the size, diversity of cases (e.g., supine vs. erect exams, if this variation was used), and how positioning quality was labelled.**

**Response: We appreciate your suggestion. In the Data Source section(Lines 104-110), we have added descriptions to emphasize that all images are strictly PA projections, with no lateral or decubitus variations, reflecting real clinical scenarios. Additionally, in the Label Classification section(Lines 131-143), we have provided a detailed description of the dataset's classification criteria, including the precise size and composition of the dataset, and have further explained the specific categories encompassed by "Others" (including non-standard postures).**

**3.Clinical Validation: It would strengthen the paper to include or suggest a pathway for clinical validation, especially if the model will be used in actual clinical radiography settings.**

**Response: We agree on the necessity of clinical validation. On one hand, our test dataset is based on real clinical chest X-ray shooting scenarios and has never appeared in the training or validation sets, meaning the test set itself is an actual clinical chest X-ray shooting scenario test. On the other hand, the model is designed to prepare for automated shooting under human supervision. Further clinical testing must be combined with the equipment currently under development to have clinical value in the next steps.**

**4.Discussion of Limitations: While the model's performance is promising, a more detailed discussion of its current limitations and generalisability to other institutions or equipment types would enhance transparency.**

**Response: We have taken your feedback into consideration and have rewritten a section on the study's limitations. Additionally, we have integrated the content from the final paragraph of the discussion that looked towards future prospects(Lines 360-376). We have also changed the subheading to "Limitations and Future Prospects."**

**Overall, the manuscript makes a valuable contribution to the field of AI in radiography and could be further strengthened by addressing the above points. I encourage the authors to refine the manuscript based on the below focused feedback and look forward to seeing this work contribute to improved imaging practices in clinical environments.**

**Introduction: line 56 -57**

**Suggestion: In or before this paragraph, include a brief Hx of the ResNet architecture and how it has made a contribution to medical imaging advancements**

**Response: We appreciate the reviewers' inquiry regarding the choice of model. The earlier version mentioned the value of ResNet in the introduction. Based on the first round of revisions, a reviewer suggested that we discuss ResNet18 in the model selection subsection of the Materials and Methods section(Lines 166-179). Considering the recommendations from both reviewers, we have added a detailed rationale for selecting ResNet18 in the Materials and Methods section, including its computational efficiency and comparison with more complex models, to enhance the methodological justification.**

**Study design: line 96 -97**

**Should this not be in the past tense if it was a completed action?**

**Response: We appreciate the reviewer’s correction regarding the use of tense. We have revised the sentences in the study design section to past tense to accurately reflect the completed research activities(Lines 158-159).**

**Materials and methods: line 155**

**Include what led to the selection of the ResNet18 specifically vs. ResNet34 or other architectures - I am not sure if I have missed in in the manuscript.**

**Response: Following your suggestion, we have not only added a discussion on the advantages of ResNet18 in the model selection section(Lines 166-179) but also explained why ResNet34/50 was not used: (1) The parameter count of ResNet18 (11.7M vs 21.8M/25.6M) is more suitable for real-time systems; (2) Based on previous experience, the increased network complexity in ResNet34/50 often leads to model overfitting.**

**In text and Table data**

**Should this not align with what you have in text because elsewhere there’s spacing or formatting variation (e.g., “Model A”, “ModelA”). In some places you have model and in others modle**

**Response: Thank you for your thorough review. We have standardized the usage to "Model A/B" throughout the text and corrected the spelling error "Modle" in the table titles and the main text.**

**Discussion: line 251**

**Include a limitations subsection**

**Response: We have taken your feedback into consideration and have rewritten a section on the study's limitations. Additionally, we have integrated the content from the final paragraph of the discussion that looked towards future prospects. We have also changed the subheading to "Limitations and Future Prospects."(Lines 360-376)**

**Under future works and outlooks- line 338 Was this not a limitation?**

**Response: We have taken your feedback into consideration and have rewritten a section on the study's limitations. Additionally, we have integrated the content from the final paragraph of the discussion that looked towards future prospects. We have also changed the subheading to "Limitations and Future Prospects."(Lines 360-376)**

**Conclusion:**

**Reword to include broader implications or future integration with the hospital workflows.**

**Response: You made a very valid point. Following your suggestion, we have expanded the discussion on future application scenarios, including the integration of the model into fully automated DR examination center equipment, as well as optimizing resource allocation in special situations such as pandemics and battlefields.(Lines 385-388)**

**Reviewer #4: The objective of this manuscript is to develop an AI system to precisely determine the spatial relationship between the X-ray detector and the patient during chest radiography. While this is a relevant and clinically significant goal, I have several concerns and suggestions regarding the proposed methodology and the presentation of the work.**

**1. The manuscript claims to develop an AI system, but it primarily employs relatively simple or already established models. The methodological novelty or technical innovation needs to be clearly demonstrated. A detailed explanation or justification for the choice of models, including an analysis of the model architecture, theoretical basis, and rationale for selecting specific AI algorithms, is essential. In high-stakes medical applications, models require extensive validation, tuning, and interpretability to be considered reliable.**

**Response: Thank you for your attention to the methodological innovations in this paper. In response to your feedback, we have enhanced the explanation of our innovations and made the following revisions: In the Materials and Methods section (Lines 166-179), we emphasize our pioneering work in applying the five-category ResNet18 model for real-time posture assessment during chest X-ray imaging. Previous studies employed conventional CNN models with binary classification. We selected ResNet18 because it has a lower parameter count than previous models, meeting the current need for real-time feedback. Moreover, its pre-trained weights help overcome the vanishing gradient problem and accelerate model convergence. This represents the first exploration into real-time posture assessment for chest X-rays, and the results have been quite successful, with improved accuracy compared to previous studies.**

**Additionally, we believe the clinical innovation of this paper surpasses its technological innovation. The significance of training this model lies in its ability to work alongside automated equipment to achieve fully automated chest X-ray imaging with human supervision. This approach can be expanded to handle large-scale chest X-ray screening tasks, reducing the workload of radiologic technologists. Furthermore, it can be authorized to optimize resource allocation in special scenarios such as wartime or pandemics.**

**2. The Methods, Results, and Discussion sections are described in broad terms and lack the scientific precision expected in a research manuscript. It would be helpful if the methods were detailed more thoroughly to improve the reproducibility and credibility of the work. Similarly, the Results and Discussion should be supported by clear descriptions, thorough analysis, and well-structured interpretation.**

**Response: You made a very valid point, and we have further refined the scientific accuracy of the manuscript. We have detailed the data classification criteria in the Materials and Methods section (Lines 131-141), providing a thorough explanation of the "Others" category, which is a state that needs to be determined for the subsequent development of fully automated equipment. Additionally, we have enhanced the Results section by adding explanations for figures and tables. In the Discussion section, we have included a detailed discussion on model selection and the limitations of the study, and integrated the title of the concluding subsection of the discussion paragraphs.**

**3. The data source section lacks clarity. For example, in line 113, the term “images deemed irrelevant” is vague—please specify the exclusion criteria. The manuscript should also clarify the standards for data inclusion, exclusion, and cleaning. Additionally, the five classification categories are not clearly defined. Similar ambiguities appear throughout the manuscript and should be addressed.**

**Response: Thank you for your attention to the data sources. In accordance with your insightful comments, we have refined the data classification criteria in the Materials and Methods section (Lines 131-141), providing a detailed explanation of the "Others" category, which is a state that needs to be determined for the subsequent development of fully automated equipment. The states of "Too High," "Too Low," "Suitable," and "No one" have been clearly defined according to the Chinese standard literature [16] in the Materials and Methods section (Lines 107-110).The specific standards are as follows: "Too High" (detector upper edge is more than 2 cm above the standard position), "Too Low" (detector upper edge is more than 2 cm below the standard position), "Suitable" (detector upper edge is within 2 cm above or below the standard position, which is approximately 3 cm above the shoulder peaks), "No one" (no patient in the shooting area), and "Others" (The "Others" category mainly refers to situations where the patient appears in the detector area but has not reached the predefined central area or assumed the standard posture. This classification serves as a basis for developing a fully automated process for PA chest X-rays. In the subsequent development of the automatic shooting device, if the system determines an "Others" status, it will not adjust the detector's height but will first prompt the patient to assume the standard position before reassessing).**

**4. In line 146, the manuscript mentions reviewing numerous journals but cites only a few references from the same source. I recommend expanding the literature review to include a broader and more diverse range of high-impact references. Moreover, the novelty of the proposed model remains unclear—it would be helpful if the manuscript clarified how it differs from existing approaches, including reference [17].**

**Response: Based on your suggestions, we have made the following revisions:**

**(1)We have indeed reviewed multiple journals, and it was an oversight on our part not to include previously mentioned references in the Introduction again. Following your advice, we have added citations to that section. However, as of now, the only study closely related to the depth of our work and suitable for comparison is reference [17].**

**(2)Regarding the innovation or novelty of the article, we have added explanations in the Materials and Methods section (Lines 166-179) and the Discussion section (Lines 305-312). These additions highlight the pioneering exploration of this task, the improvement in accuracy, the balanced fulfillment of lightweight real-time performance and accuracy, and the significant clinical implications.**

**5. Please include a more thorough explanation of how the dataset was divided into training, validation, and test sets. If cross-validation or folds were used, a clear description of the process would improve understanding.**

**Response: Thank you for your keen attention to data classification. To detail the creation of the training, validation, and test sets, as well as the specific parameters of cross-validation, we have specifically created clear charts (figures 2 and 3) to explain this to the readers, ensuring that the work can be clearly reproduced.**

**6. If there are existing published methods that address similar problems, it would be helpful to include comparisons.**

**Response: You are absolutely right. We considered this at the beginning of the experimental design. Model A is a reproduction of previous research, as referenced in [17], and can be considered the control group. The results show that Model A is more helpful in terms of interpretability, but it is inferior to the experimental group Model B in terms of being lightweight, accuracy, and ease of training.**

**7. Given the clinical implications, I suggest a more comprehensive evaluation strategy. Referring to earlier peer-reviewed studies that have applied validated techniques (before 2022) could strengthen the validation.**

**Response: We agree on the necessity of clinical validation. On one hand, our test dataset is based on real clinical chest X-ray scenarios, which have never appeared in the training or validation sets. This means the test set itself is a real clinical chest X-ray scenario test, and this testing method is a conventional approach used in similar peer-reviewed studies. On the other hand, the design of this model is intended to prepare for automated imaging under human supervision. Further clinical testing must be combined with the currently developing equipment to realize its clinical value in subsequent stages.**

**8. Is there any limitations of the proposed approach?**

**Response: Based on your comments, we have rewritten the section on the study's limitations. Additionally, we have integrated the content on future prospects from the last paragraph of the discussion. We also changed the subtitle to "Limitations and Future Prospects." (Lines 360-376)**

**9. The manuscript would benefit from careful language editing to enhance clarity and professionalism. For instance, the term “take” in the short title is unclear. Additionally, the phrase “daily chest X-ray” in the abstract, does it refer to routine imaging? Clarification would be helpful.**

**Response: Based on your feedback, we have re-examined the language and grammar issues in the article and made the following modifications:**

**(1) Removed the word "take" and rewrote the short title.**

**(2) Changed "daily chest X-ray" to "Routine clinical chest X-ray."**

**(3) Listed the full terms for abbreviations like "DR" and "CR" upon first mention.**

**(4) Unified the representation of Model A and Model B.**

**(5) Eliminated unnecessary spaces and commas.**

**10. Figures and tables should be clearly labeled and thoroughly explained.**

**Response: Following your suggestions, we have enhanced and refined the captions and legends for all figures and tables and updated the list at the end of the document.**

**Thank you for your patience and thorough review. Any further questions can be directed to jiangbo@csu.edu.cn.**

**Best regards,**

**Your closest Situ**

**July 30, 2025**
